# An Updated Algorithm for Estimation of Pesticide Exposure Intensity in the Agricultural Health Study

**DOI:** 10.3390/ijerph8124608

**Published:** 2011-12-12

**Authors:** Joseph Coble, Kent W. Thomas, Cynthia J. Hines, Jane A. Hoppin, Mustafa Dosemeci, Brian Curwin, Jay H. Lubin, Laura E. Beane Freeman, Aaron Blair, Dale P. Sandler, Michael C. R. Alavanja

**Affiliations:** 1 Occupational and Environmental Epidemiology Branch, Division of Cancer Epidemiology and Genetics, National Cancer Institute, NIH/DHHS, 1620 Executive Blvd., Rockville, MD 20892, USA; Email: coble.joe@dol.gov (J.C.); dosemecia@ninds.nih.gov (M.D.); freemala@mail.nih.gov (L.E.B.F.); blaira@exchange.nih.gov (A.B.); 2 National Exposure Research Laboratory, Office of Research and Development, U.S. Environmental Protection Agency, MD-E205-04, Research Triangle Park, NC 27711, USA; Email: thomas.kent@epa.gov; 3 National Institute for Occupational Safety and Health, 4676 Columbia Parkway R-14, Cincinnati, OH 45226, USA; Email: cjh8@cdc.gov (C.J.H.); bic4@cdc.gov (B.C.); 4 Epidemiology Branch, National Institute of Environmental Health Sciences, NIH/DHHS, MD-A3-05, P.O. Box 12233, Research Triangle Park, NC 27711, USA; Email: hoppin1@niehs.nih.gov (J.A.H.); sandler@niehs.nih.gov (D.P.S.); 5 Biostatistics Branch, Division of Cancer Epidemiology and Genetics, National Cancer Institute, NIH/DHHS, 1620 Executive Blvd., Rockville, MD 20892, USA; Email: lubinj@exchange.nih.gov

**Keywords:** pesticides, exposure algorithm, epidemiology, 2,4-D, chlorpyrifos, captan

## Abstract

An algorithm developed to estimate pesticide exposure intensity for use in epidemiologic analyses was revised based on data from two exposure monitoring studies. In the first study, we estimated relative exposure intensity based on the results of measurements taken during the application of the herbicide 2,4-dichlorophenoxyacetic acid (2,4-D) (n = 88) and the insecticide chlorpyrifos (n = 17). Modifications to the algorithm weighting factors were based on geometric means (GM) of post-application urine concentrations for applicators grouped by application method and use of chemically-resistant (CR) gloves. Measurement data from a second study were also used to evaluate relative exposure levels associated with airblast as compared to hand spray application methods. Algorithm modifications included an increase in the exposure reduction factor for use of CR gloves from 40% to 60%, an increase in the application method weight for boom spray relative to in-furrow and for air blast relative to hand spray, and a decrease in the weight for mixing relative to the new weights assigned for application methods. The weighting factors for the revised algorithm now incorporate exposure measurements taken on Agricultural Health Study (AHS) participants for the application methods and personal protective equipment (PPE) commonly reported by study participants.

## 1. Introduction

The risk of adverse health effects associated with long-term exposure to pesticides is difficult to assess in epidemiologic studies due to various limitations that have been summarized in the literature [1]. A major challenge has been the development of reliable methods to estimate the duration and intensity of exposure to pesticides in large studies in which the direct measurement of exposure to all participants is not feasible [2,3,4]. The Agricultural Health Study (AHS) is a prospective cohort study of 57,310 licensed private and commercial pesticide applicators, primarily farmers, and 32,345 spouses, designed to investigate health effects associated with pesticides and other agricultural exposures [5]. At enrollment, pesticide applicators completed self-administered questionnaires to provide information on lifetime frequency and duration of use for 50 specific pesticides, frequency of mixing or loading of pesticides, application methods, frequency of repair of pesticide application equipment and use of personal protective equipment (PPE). To utilize the information collected on the enrollment questionnaire to estimate exposure intensity, we previously developed an exposure algorithm (denoted version 1) [6]. As described by Dosemeci *et al*., the weighting factors in the algorithm were developed based primarily on expert judgment using published studies on pesticide exposure from the world’s literature, including information from the Pesticide Handlers Exposure Database (PHED) [7]. The weighting factors (*i.e.*, numerical values), when used in the algorithm, convert categorical responses to specific questions from the enrollment questionnaire from each applicator into a relative exposure intensity score. The exposure intensity scores are multiplied by frequency and duration of use as reported on the questionnaire to calculate lifetime intensity-weighted days of pesticide use for epidemiological analyses. 

The AHS algorithm has four variables that were combined as follows: 

                 Exposure Intensity Score = ([MIX] + [APPLY] + [REPAIR]) × [PPE]) 

where [MIX] represents exposure from mixing and loading operations prior to application, [APPLY] represents exposure from applying pesticides, [REPAIR] represents exposure from contact with contaminated surfaces during the repair of pesticide application equipment, and [PPE] represents an exposure reduction factor to account for use of PPE. 

The reliability of the version 1 algorithm intensity scores for correctly rank ordering various application scenarios has been evaluated based on four field monitoring studies; (1) a study among Canadian farmers [8], (2) a study among Minnesota and South Carolina pesticides applicators [9], (3) the AHS Pesticide Exposure Study (AHS/PES) [10,11], and (4) the AHS Orchard Fungicide Exposure Study (AHS/OFES) [12,13,14]. Because the two field monitoring studies conducted on subgroups of AHS applicators after the algorithm was developed offered AHS-specific, quantitative measurements for various application characteristics, we used these data, in conjunction with the world’s literature and PHED, to modify the algorithm weights, thereby reducing the need to rely exclusively on measurement data external to the cohort. The field monitoring results, in general, confirmed the underlying premise of the algorithm; *i.e.*, that algorithm scores based primarily on application method and the use of personal protective equipment can be used to identify applicators most likely to have encountered higher pesticide exposure levels, and thereby serve as an effective surrogate for exposure intensity. Nonetheless, the exposure measurements suggest that some modifications to the algorithm weights (denoted version 2) could be made that would improve agreement with the results of these field monitoring studies, and thereby potentially reduce exposure misclassification inherent in the use of any algorithm. 

In the AHS/PES, we selected 2,4-D and chlorpyrifos because 2,4-D is one of the most important agricultural and residential herbicides and chlorpyrifos is one of the most important agricultural insecticides. In addition, the pharmacokinetics of these chemicals are relatively well understood. Both chemicals are widely used by AHS cohort members. Similarly, the AHS/OFES measured captan, the second most frequently used fungicide in the AHS. These studies included some of the most frequently used application methods in the cohort.

Measurement results from the AHS field studies were used to examine relative differences in urinary biomarker concentrations associated with the algorithm exposure variables. These comparisons enabled us to modify the algorithm weights using AHS-derived field study data while still relying on information from the literature and PHED for algorithm weights, particularly where AHS-specific field data was lacking. Decisions on changing any algorithm weights were based on the field study data in combination with the body of information from the literature and PHED. In addition, we re-scaled the algorithm scores and assigned weights for application methods reported by cohort members in follow-up questionnaires but not in the enrollment questionnaire. These enhanced algorithm weights provide the basis for updated exposure intensity scores currently used in AHS epidemiological analyses. 

## 2. Field Studies

The methodology and measurement results for the AHS/PES have been previously described in detail [10]. The AHS/PES study selected applicators who reported agricultural use of 2,4-D or chlorpyrifos on the AHS Phase II questionnaire in 22 counties in eastern Iowa and 22 counties from eastern and central North Carolina. The AHS/PES study collected pre- and post-application urine samples, as well as hand wipe, body patch and personal air samples [10]. The post-application urine sample was a composite sample collected from the beginning of a monitored application through the first morning void the next day. Results from 68 applicators for 88 applications of 2,4-D and from 16 applicators for 17 applications of chlorpyrifos were used in this analysis. Where repeat measurements were made on an individual, the interval between measurements ranged from one week to 14 months; however, as described previously [10], several applicators reported using the chemical in an unmonitored application within four days prior to the monitored application. All 2,4-D broadcast spray applications (N = 46) were made with tractor-mounted boom sprayers except for one truck-mounted boom sprayer and one highboy application and were grouped into a ‘boom spray’ category for this analysis. Hand spray applications of 2,4-D (N = 42) were made using vehicle-mounted or portable sprayers. In three applications, both boom spray and hand spray methods were used; these applications were placed in the hand-spray group for analysis. Chlorpyrifos application methods included in-furrow or banded applications of a granular formulation (n = 13), and spray applications of a liquid formulation by boom (N = 3) and airblast (N = 1) sprayers. For our purposes, we classified chlorpyrifos applications as either boom spray/liquid or in-furrow/granular. Applicators personally mixed and/or loaded pesticide products, except for five cases where someone else performed the mixing/loading. The AHS/OFES selected all orchard farmers in Iowa and North Carolina who reported growing apples or peaches on the AHS Phase 2 questionnaire [12]. The AHS/OFES measured captan, a fungicide, for 74 applicators on 144 days when it was applied to orchards using either hand spray or air blast methods [12,13,14]. Measurements included personal air, hand rinse and dermal patch samples, as well as pre-application and 24-h post-application urine samples. Both field studies were observational in design. Applicators in these studies followed their usual procedures with regard to mixing and application procedures, duration of the application, total amount of pesticide applied, and type of PPE worn during different phases of the application process. Information pertaining to the algorithm variables was obtained from observations by study personnel and, for the AHS/PES, using interviewer-administered questionnaires. AHS research was reviewed and approved as applicable by Institutional Review Boards at the National Cancer Institute, the University of Iowa, Battelle; RTI International, and the National Institute for Occupational Safety and Health. 

### 2.1. Statistical Analysis

Arithmetic means, geometric means (GM) and geometric standard deviations (GSD) of post-application urine concentrations for AHS/PES applicators were calculated for application method and use of chemical-resistant or other waterproof gloves (referred to as CR gloves). We used a two-way analysis of variance procedure among study participants (GLM Procedure, SAS version 9.1, Cary, NC, USA) to evaluate whether CR-glove use or application method significantly affected the urine concentrations of the measured analyte, when controlling for the other factor. Urine concentrations were log-transformed to account for right skewed data. 

We calculated the ratios of the GM’s to evaluate the relative exposure intensity for (1) for boom spray compared to an in-furrow/granular application method and (2) the reduction in post-application urine concentrations attributable to glove use. Spearman correlation coefficients were calculated between version 2 *vs.* version 1 algorithm scores for measurements of 2,4-D and chlorpyrifos in post-application urines. 

To provide a secondary method to evaluate the revised weighting factors, we fitted a nonlinear regression model to assess the joint influence of the algorithm variables on post-application urine concentrations (Y) in μg/L: 

      Y = {α_0_ + α_1_ Mix + α_2_ Method + α_3_ Repair} × {1 − (β_1_ Gloves) – (β_2_ PPE other)} (1)

where α_0_ represented the urinary concentration at the referent level of all factors, where α_1_, α_2_ and α_3_ parameters represented the increase in Y for mixing (1 = yes, 0 = no), use of hand spray (method = 1) or boom spray (method = 0) for 2,4-D, or boom spray (method = 1) or in-furrow (method = 0) for chlorpyrifos, and repairing equipment (1 = yes, 0 = no), respectively, and where β_1_ and β_2_ parameters represented the reduction factors for use of CR gloves (1 = yes, 0 = no) and/or other PPE (1 = yes, 0 = no), respectively. We then compared the predicted values from the model to the algorithm scores. Because the regression coefficients were pesticide specific and based on relatively limited data in many of the exposure scenarios, we did not directly use the parameter estimates as weights, but rather to jointly assess the relative influences of the variables. 

To evaluate the extent to which algorithm scores could be used to categorize applicators into exposure groups, we divided the 2,4-D applicators into three groups by algorithm score (<50, 50–100, >100), computed summary statistics, and conducted a nonparamteric test for trends based on rankings using the Stata nptrend command, an extension of the Wilcoxon rank-sum test. Due to a smaller number of applications and limited range of scores, the chlorpyrifos data were divided into two groups using a cut-point of 50. 

## 3. Results and Discussion

### 3.1. Use of CR Gloves

CR glove use was associated with a significant difference in urinary 2,4-D GM levels overall, when controlling for application method (p < 0.0001). Among 2,4-D applicators who wore CR gloves, GMs of the post-application urine concentrations were 75% and 72% lower for boom (14 µg/L *vs.* 55 µg/L) and hand spray (23 µg/L *vs.* 81 µg/L) applicators, respectively, compared with those who did not wear CR gloves (Table 1). 

Among chlorpyrifos applicators, the GMs of 3,5,6-trichloro-2-pyridinol (TCPy) post-application urine concentrations were 50% and 56% lower with CR glove use for in-furrow (granular formulation) and boom spray (liquid formulation) application, respectively, (GM = 6 µg/L and GM = 14 μg/L) compared with no glove use (12 µg/L and 32 μg/L). While CR glove use was associated with lower GM TCPy levels, the results were not statistically significant (p = 0.084) when we controlled for application method.

Based on a reduction of 72% to 75% among the 2,4-D applicators, and of 50% to 56% among the chlorpyrifos applicators, the reduction factor for use of CR gloves was increased from 40% in the version 1 algorithm to 60% in version 2. 

### 3.2. Application Method

Among 2,4-D applicators, the GMs for hand spray applicators were 1.6 times and 1.5 times higher than for boom spray applicators who did (23 μg/L *vs.* 14 μg/L) and did not wear CR gloves (81 μg/L *vs.* 55 μg/L) (Table 1). Although 2,4-D levels for hand spray were higher than for boom spray, the difference was not statistically significant after controlling for glove use (p = 0.092). 

For chlorpyrifos applicators, the GMs for boom spray applicators were 2.3 and 2.7 times higher than for in-furrow applicators for those who did (14 μg/L *vs.* 6 μg/L) and did not (32 μg/L *vs.* 12 μg/L) wear CR gloves, respectively. Although boom spray results are based on only four observations, when we controlled for CR glove use, we observed a significantly higher GM concentration of TCPy associated with boom spraying *vs.* in-furrow application (p = 0.014). 

Based on the ratio of the GM’s by application method, we decided to increase the weighting factor for boom spray, thereby reducing the relative difference with hand spray from version 1 (*i.e.*, 3:9) compared to version 2 (*i.e.*, 40:90); and increasing the relative difference with in-furrow from version 1 (*i.e.*, 3:2) compared with version 2 (*i.e.*, 40:20). 

**Table 1:** Post-application urine concentrations (µg/L) grouped by application method and CR glove use for 2,4-D ^1^ (N = 88) and chlorpyrifos ^2^ (N = 17) applications.

Application Method	CR Glove Use	N	AM	GM	GSD	CR Glove Use ^3^	Application Method ^3^
2,4-D							
Boom Spray	Yes	32	27	14	3.1	P < 0.0001	P = 0.092
	No	14	91	55	3.0		
Hand Spray	Yes	21	48	23	3.3		
	No	21	200	81	4.9		
Chlorpyrifos							
In-furrow (granular)	Yes	7	8	6	1.8	P = 0.084	P = 0.014
	No	6	14	12	1.8		
Boom Spray(liquid)	Yes	2	14	14	1.3		
	No	2	47	32	3.6		

^1^ 2,4-D measured as a urinary biomarker for 2,4-D. ^2^ TCPy measured as a urinary biomarker for chlorpyrifos. ^3^ P values from two-way analysis of variance using (independent variables: glove use and application method). Abbreviations: AM = arithmetic mean; CR = chemically-resistant; GM = geometric mean; GSD = geometric standard deviation; N = number of application days monitored.

In the version 1 algorithm, hand spray and air blast had the same weight (*i.e.*, 9); however, among captan applicators the AHS/OFES detected *cis*-1,2,3,6-tetrahydrophalimide (THPI), a metabolite of captan, in 77% of urine samples from 79 air blast applications (range, <1.7 to 32.0 μg/L) compared with 41% of samples from 59 hand spray applications (range, <1.7 to 29.9 μg/L) [13]. The percent detected was approximately 88% higher for airblast compared to hand spray. Due to the high percentage of non-detects among hand spray applicators, we did not estimate a GM; however, we decided to increase the weighting factor for airblast to 150 so that it would be substantially higher than the weighting factor of 90 for hand spray in the version 2 algorithm (67% higher). The effect of this change was that an airblast applicator would be assigned a higher weight score (*i.e.*, 150) than a hand spray applicator, even if the hand spray operator both mixed/loaded and applied (*i.e.*, 50 + 90 = 140). Because the information from the captan study used in this assessment was based only on the percentage of detectable measurements for different application methods, no statistical analyses were performed for captan. 

### 3.3. Version 2 Algorithm Weights

The version 2 algorithm retained the same four variables as version 1 because these variables were *a priori* determinants of interest and therefore had been collected for all applicators at enrollment. We made the following modifications to version 2: (1) rescaled the range of scores by a factor of 10; (2) increased the reduction for use of CR gloves; (3) increased the weights for boom spray and air blast application methods; and (4) reduced the weight for mixing (Table 2). 

In the version 1 algorithm, intensity scores ranged from 0.1 to 20, with scores that included decimal values. To use only integers with a minimum value of 1, the version 2 algorithm weights were re-scaled by a factor of 10, so version 2 intensity scores range from 1 to 220. Rescaling was done primarily for convenience and had no effect of the relative ranking by algorithm score. 

**Table 2:** AHS Pesticide Exposure Algorithm Weighting Factors. Algorithm Intensity Score = (MIX + APPLY + REPAIR) × PPE.

**MIX**	**Version 1**	**Version 2**
Did Not Mix	0	0
Mix <50% of the time	3	20
Mix >50% of the time	9	50
**REPAIR**	**Version 1**	**Version 2**
No	0	0
Yes	2	20
**APPLICATION METHODS**	**Version 1**	**Version 2**
Air blast	9	150
Hand Spray	9	90
Mist Blower Or Fogger	9	90
Fog Or Mist Animals	9	90
Greenhouse Sprayer	9	90
Pour Fumigant From Bucket	9	90
Powder Duster	9	90
**MIX**	**Version 1**	**Version 2**
Backpack Sprayer	8	80
Dust Animals	7	70
Pour On Animals	7	70
Garden Hose	None	50
Hand Held Squeeze Or Squirt Bottle	None	50
Watering Can/Sprinkling Can	None	50
Soil Injected Or Drilled	4	40
Spray Over Rows	4	40
Boom On Tractor	3	40
Broadcast Application	3	40
Personally Applied To Seed	2	40
Banded/Directed Spray (liquid)	2	30
Banded Application (granular)	2	20
Gas Canister	2	20
Hang Pest Strips In Barn	2	20
In-Furrow	2	20
Incorporated	2	20
Inject Animals	2	20
Seed Treatment	1	20
Hand Spreader Or Push Spreader	None	20
Planter Box	None	20
Aerial	1	10
**PPE REDUCTION**	**Version 1**	**Version 2**
Chemical Resistant or Rubber Gloves	40%	60%
Cartridge Respirator, Tyvek Coveralls	30% for use of 1 or more	10% each with max of 30%
Face Shield, Goggles, Boots, Apron, Other	20% for use of 1 or more	
Fabric/leather gloves	20%	none

^1^ None indicates methods for which a version 1 weighting factor was not assigned

In the version 2 algorithm, the protection factor for glove use was increased from 40% to 60%. The increase was based on comparison of the GM urine concentrations for CR glove use relative to no CR glove use that ranged from 50% to 75% (Table 1). Data from the AHS/PES and the PHED data base generally demonstrate that personal protective equipment rarely reduce the amount of exposure expected from a particular exposure scenario more than 90%. With the protective factor for CR rubber gloves increasing to 60%, we have assigned a further increase in protection with each additional piece of equipment, including coveralls, respirators, face shield/goggles and CR boots, up to 90% protection. We could not clearly distinguish between the levels of protection afforded by the various types of equipment so we assigned a 10 % reduction for each piece of equipment up to a maximum of 30%.

The enrollment questionnaire asked about use of “chemically" resistant gloves (for example, neoprene or nitrile gloves), and because we could not distinguish between different types of CR gloves based on the enrollment questionnaire, we assigned the same reduction for rubber, waterproof or disposable latex gloves as for CR gloves. The version 1 algorithm included a 20% reduction use of fabric/leather gloves. Data from our monitoring study AHS/PES study, however, did not support treating fabric/leather gloves as protective, and therefore, the version 2 algorithm does not assign any reduction in exposure for their use. 

We increased the weight for boom spray application from 3 (on version 1 scale) to 40 (on version 2 scale) while retaining the banded/in-furrow application method weight at 2 (20 on the version 2 scale) to reflect the approximately 2-fold exposure difference observed in the chlorpyrifos data. Based on the detection frequency difference of THPI in the AHS/OFES, we increased the air blast application weight to 150 which was now 67% higher than the hand spray weight of 90. This change ensured that airblast would be the application method with the highest exposure potential under all exposure scenarios. Because post-enrollment AHS questionnaires expanded the number of application methods, we accommodated these additional methods in the version 2 algorithm by assigning weights based on similarities to previously assigned methods (Table 2).

In version 1, the weight for mixing equaled the weight for hand spray (previously the highest application method weight). In version 2, we assigned a relatively smaller weight of 50 for mixing (*versus* 90 for hand spray). This reduction increased the difference in intensity scores for applicators who both mixed and applied using different application methods. For example, version 1 scores for boom spray *versus* an in-furrow application for those who mixed were 9 (version 1 mix weight) + 3 (version 1 boom spray weight) = 12 *versus* 9 (version 1 mix weight) + 2 (version 1 in-furrow weight) = 11, respectively, a difference of less than 10%. The version 2 intensity scores were 50 (version 2 mix weight) + 40 (version 2 boom spray weight) = 90 and 50 (version 2 mix weight) + 20 (version 2 in-furrow weight) = 70, a difference of almost 30%. 

Because only five 2,4-D applicators did not personally mix or load on the morning prior to monitoring, the amount of data available to assess exposure that occurs during mixing compared with the rest of the application process was limited. The GM of the post-application urine concentrations for applicators who mixed on the morning of urine collection was ~50% higher than those who did not mix, which is somewhat lower than previously reported in the literature [6,7]. Our revised weight for mixing is now less than the weight for hand spray method, and only slightly larger than the weight for boom spray application. 

Repairing equipment increased exposure for 2,4-D applicators (GM = 34 µg/L, n = 26 who repaired *vs.* 28 µg/L, n = 62 who did not repair). Little difference was seen for chlorpyrifos (TCPy) (GM = 10 µg/L, n = 8 who repaired *vs.* 11 µg/L, n = 9 who did not repair), although the sample size was small. Given the limited data, we did not modify the algorithm weight for repair.

Spearman correlation coefficients between version 2 algorithm score and measurements of 2,4-D in post-application urine were greater than the Spearman correlation between version 1 algorithm scores and measurements of 2,4-D in post-application urine but not for chlorpyrifos (Table 3). Correlation coefficients for 2,4-D also increased for version 2 *vs*. version 1 for the hand, body and air (data not shown). Correlation coefficients were also increased for version 2 algorithm scores and measurements of chlorpyrifos on the hand and body (data not shown). Spearman correlation coefficients between version 1 and version 2 algorithm scores were very high for both 2,4-D (r = 0.95) and chlorpyrifos (0.97) applications.

**Table 3:** Spearman correlation coefficients between Version 1 algorithm scores and measurements of post-application urine 2,4-D and chlorpyrifos and modeled post-application urine concentrations for 2,4-D (N = 88) and chlorpyrifos (N = 17) and Version 2 algorithm scores with post-application urine concentrations and modeled post-application urine concentrations for 2,4-D and chlorpyrifos.

	Algorithm	
	Version 1	Version 2
2,4-D		
Version 1	1	
Version 2	0.95	1
Post-apply urine conc.	0.42	0.48
Predicted post-apply urine concentration ^1^	0.96	0.97
Chlorpyrifos ^2^		
Version 1	1	
Version 2	0.97	1
Post-apply urine conc.	0.53	0.52
Predicted post-apply urine concentration	0.52	0.59

^1^ Modeled value from a non-linear regression mode l. ^2^ TCPy measured as a urinary biomarker for chlorpyrifos.

We fitted a nonlinear model based on the algorithm formula (1) to compare the updated weights with parameter estimates from a joint analysis of all component variables simultaneously. Coefficients were in the expected direction and the application method and CR-glove PPE terms were significant (see Table 4 for parameter estimates). Use of CR gloves was statistically significant for both 2,4-D and chlorpyrifos with estimated reductions for use of gloves of 75% and 51%, respectively. Application method was also statistically significant, with higher urine concentrations for hand spray compared to boom spray for 2,4-D and for boom spray compared to in-furrow application for chlorpyrifos. For 2,4-D, the regression parameters for mix and repair were not statistically significant; however, the direction and relative magnitude of the estimates were consistent with their corresponding algorithm weights. For chlorpyrifos, all applicators mixed and applied, so the mix variable could not be evaluated and the repair variable was also not statistically significant. The predicted concentrations from the model were highly correlated with the Version 2 algorithm scores (Table 3).

**Table 4:** Nonlinear regression of post-application urine concentration on algorithm. Y = [{α_0_} + {α_1_} × mix + {α_2_} × method + {α_3_} × repair] × [1 − {β_1_} × gloves − {β_2_} × ppe_other].

2,4-D (n = 88)	R-Squared = Regression	0.36
Variable ^1^	Coefficient	P-value
Intercept α_0_	27	0.76
Mix α_1_,	58	0.53
Method α_2_	123	0.02
Repair α_3_	32	0.59
Gloves β_1_	0.75	<0.001
PPE other β_2_	0.26	0.26
Chlorpyrifos (n = 17)	R-Squared = Regression	0.77
Variable^1^	Coefficient	P-value
Intercept α_0_	8	0.22
Mix α_1_,	Na ^2^	Na ^2^
Method α_2_	33	0.006
Repair α_3_	15	0.89
Gloves β_1_	0.51	0.014
PPE other β_2_	0.21	0.59

^1^ α_0_ represented the urinary concentration at the referent level of all factors, where α_1_, α_2_ and α_3_ parameters represented the increase in Y for mixing (1 = yes, 0 = no), use of hand spray (method = 1) or boom spray (method = 0) for 2,4-D, or boom spray (method = 1) or in-furrow (method = 0) for chlorpyrifos, and repairing equipment (1 = yes, 0 = no), respectively, and where β_1_ and β_2_ parameters represented the reduction factors for use of CR gloves (1 = yes, 0 = no) and/or other PPE (1 = yes, 0 = no), respectively. ^2^ na: all participants mixed chlorpyrifos and the regression omitted the variable.

When grouped by approximate tertile of the algorithm scores, we found a statistically significant trend (p ≤ 0.01) in the post-application 2,4-D GM concentrations (Table 5). For chlorpyrifos, urine concentrations of TCPy were significantly higher among applicators with algorithm scores above 50 compared to the applicators with an algorithm score category less than 50 (p = 0.03).

**Table 5:** Arithmetic means, geometric means and geometric standard deviation of post-application urine concentrations by Version 2 algorithm score category.

2,4-D					
Category	Range	N	AM	GM	GSD
<50	12–48	40	30	15	3.2
50–100	59–90	24	78	39	3.6
>100	110–160	24	178	69	4.7
All		88	84	30	4.2
p-trend	<0.01				
Chlorpyrifos ^1^					
Category	Range	N	AM	GM	GSD
<50	24–36	9	10	8	2.1
≥50	70–110	8	22	16	2.1
All		17	11	10.6	2.3
p-trend	0.03				

^1^ TCPy measured as a urinary biomarker for chlorpyrifos. Abbreviations: AM = Arithmetic Mean, GM = geometric mean, GSD = Geometric Standard Deviation.

### 3.4. Discussion

Developing estimates of pesticide exposure intensity for large-scale cohort studies is a challenging, but critical task for exposure–response analysis. The use of simple exposure metrics, such as duration, fails to account for large differences in cumulative exposure that can occur because of the amount and concentration of active ingredients in the pesticide products applied, mixing and application methods, equipment size and design, PPE use, individual work practices and personal hygiene [2,10,11,14,15]. Measurements from the AHS/PES demonstrated substantial variability in exposure as a indicated by 2,4-D post-application urine concentrations that ranged over three orders of magnitude (1.6 to 1,040 µg/L) [10]. Moreover, substantial variability in 2,4-D and chlorpyrifos urine concentrations was observed for applicators using the same application methods, which further highlighted the difficulty in predicting individual exposure levels from questionnaire data. However, when using an algorithm with multiple variables, we found correlations for version 2 algorithm scores and urine concentrations of 0.48 for 2,4-D and 0.52 for chlorpyrifos, and increasing GMs of urine concentrations by increasing categories of algorithm score, suggesting that our algorithm captures important components of applicators' exposure intensities. 

Although we fitted a model to compare the updated algorithm weights with parameter estimates from a joint analysis of all component variables simultaneously, we did not use the coefficients from the model directly to change algorithm weight because coefficients were pesticide specific, based on relatively limited data and encompassed relatively few exposure scenarios. Nonetheless, coefficients were in the expected direction and the application method and PPE terms were significant, supporting the usefulness of the exposure algorithm.

Previous evaluations of the AHS algorithm (version 1) in both non-AHS and AHS applicators demonstrated its usefulness [8,9,10,11,12,13,14,15] in categorizing applicators into groups with significantly different average exposure levels. Coble [8] compared algorithm scores for applicators of the herbicides 2,4-D and 2-methyl-4-chlorophenoxyacetic acid (MCPA) with post-application urine concentrations and found correlations of 0.49 for 2,4-D and 0.17 for MCPA, suggesting the potential for herbicide-specific differences. In Minnesota and South Carolina applicators [9], correlation coefficients for algorithm scores and urinary concentrations were 0.47 for glyphosate, 0.45 for 2,4-D and 0.42 for liquid chlorpyrifos, but 0.12 for any chlorpyrifos (*i.e.*, granular or liquid). In the AHS/OFES study, version 1 algorithm scores were predictive of dermal thigh patch levels, but not the post-application urine, hand, or air concentrations for captan [13]. An assessment of the version 1 algorithm within the AHS/PES data showed that algorithm scores and urinary concentrations were significantly correlated for both 2,4-D (r = 0.42) and chlorpyrifos (r = 0.53) [11]. Information collected from epidemiologic questionnaires spanning a working life-time necessarily constrains the number and type of variables that we can include in any exposure algorithm. We were thus unable to incorporate additional factors that may be predictive of exposure, such as, amount of active ingredient applied, application duration, number of tanks mixed/loaded, number of acres treated, formulation, spills or splashes and dermal contact with sprayed vegetation. These and other factors, including personal hygiene and other differences in work practices, increase uncertainties in exposure characterization; however, algorithm intensity scores in the AHS are not used alone; they are always applied to an estimate of lifetime days of use for each pesticide which serves as a measure of the relative amount of use in a lifetime. 

Information about several commonly used application methods was obtained using the enrollment questionnaire. Additional application methods used by members of the cohort have been identified in subsequent follow-up data collections. Robust exposure measurement data were not available for assigning algorithm score weights for these methods, so scores previously developed for similar methods were assigned. The uncertainty in these assignments is a limitation of the updated algorithm. 

Because liquid chlorpyrifos was always applied by spraying and granular chlorpyrifos was always applied using banded or in-furrow methods in the AHS/PES study, we could not distinguish between application method or formulation type. Both dermal measurements and urine concentrations were higher for liquid spray applications than for in-furrow granular applications. Formulation type was not included in the algorithm because it was not collected in the enrollment questionnaire.

While exposure levels varied by chemical, we lacked sufficient measurement data on determinants of exposure for multiple pesticides under different application scenarios to develop pesticide-specific weights, and therefore algorithm weights apply to all pesticides. In addition, differences in absorption, metabolism and excretion rates for different pesticides and tissue-specific effects did not allow algorithm intensity scores to estimate internal doses directly. Nonetheless, it was clear from the results that the algorithm scores, on average, provided an indicator of exposure intensity for applicators using the most commonly reported application methods in the AHS cohort. Epidemiologic analyses of the AHS cohort have used the algorithm score (version 1) extensively as a measure of exposure intensity (http://aghealth.nci.nih.gov/).

Both version 1 and 2 of the algorithm are based on an extensive review of the world’s literature and the use of the Pesticide Handlers Exposure Database (PHED) which included many different chemicals (6). With the addition of revised algorithm weights derived from the two field studies within the AHS we were able to adjust the weights to account for local variations in farming practices and conditions. We judge version 2 to be superior to version 1 but the correlations between version 1 and version 2 are high r = 0.95 for 2,4-D and r = 0.97 for chlorpyrifos. This demonstrates that local conditions and characteristics can have some influence on algorithm weights, although the degree of influence is not substantial. The revised algorithm (version 2) will be used in future AHS epidemiologic analyses. 

## 4. Conclusions

Revised weighting factors in a pesticide exposure intensity algorithm were developed for use in epidemiologic analyses for the Agricultural Health Study by using exposure monitoring data from two monitoring substudies in combination with the world’s exposure literature and PHED. 
